# Comparative study of *in vitro* digestibility and antioxidant activities of whey from different milks

**DOI:** 10.3389/fnut.2026.1896587

**Published:** 2026-10-14

**Authors:** Meriem Ben Yagoub, Zeineb Jrad, Olfa Oussaeif, Mohamed Hammadi, Samia Azabou, Halima El Hatmi

**Affiliations:** 1 Livestock and Wildlife Laboratory LR16IRA04, Arid Land Institute of Medenine, University of Gabes, Medenine, Tunisia; 2 Food Department, Higher Institute of Applied Biology of Medenine, University of Gabes, Medenine, Tunisia; 3 Analytical, Valorization and Food Safety Laboratory, National Engineering School of Sfax, Sfax, Tunisia

**Keywords:** antioxidant activities, camel milk, cow milk, ewe milk, gastro-intestinal digestion, goat milk, mare milk, whey

## Abstract

**Introduction:**

Whey proteins, a protein-rich by-product of milk, contribute to various biological benefits. These bioactivities play an important role in human health and can be enhanced when whey proteins are digested, yet the digestion of whey proteins from different animal species under standardized protocols of *in vitro* digestion and its effect on antioxidant activity remain insufficiently characterized. This work aims to compare the digestibility and antioxidant activities of whey from the milk of different animal species: cameline, bovine, ovine, caprine, and equine.

**Methods:**

Milk samples from the five species were coagulated to obtain whey. The whey proteins were digested with pepsin and pancreatin in an *in vitro* process that mimics digestion in the gastrointestinal tract. Physicochemical parameters, protein profiles (SDS‑PAGE), peptide concentration, degree of hydrolysis (DH), and antioxidant activities were analyzed to assess inter‑species differences.

**Results and discussion:**

After milk coagulation, a slight increase in pH values was observed in all whey samples. The maximum peptide concentration was observed in the digested ewe whey, while digested mare whey showed the lowest. Camel whey has the highest content of sodium, potassium, zinc and iron, while goat whey has the highest concentration of copper and ewe whey has the highest concentration of calcium. The SDS-PAGE profiles showed that all five milk types had an identical protein profile, with the exception of camel whey, which was characterised by the absence of *β*-lactoglobulin (*β*-Lg) and the presence of two different proteins camel whey basic protein (CWBP) and Peptidoglycan recognition protein (PGRP). The highest antioxidant activity was found in ewe whey. The sensitivity of whey proteins to hydrolysis varies between milk types. Correlation analysis indicated that the DH was significantly positively correlated with peptide concentrations (*r* = 0.998, *p* < 0.0001), indicating that increased proteolysis facilitates peptide liberation. Simulated gastrointestinal digestion produced peptides exhibiting antioxidant properties across all five species, reinforcing the notion that whey hydrolysates offer a valuable avenue for enhancing this by-product.

**Conclusion:**

These findings highlight the nutritional and functional diversity of whey proteins among animal species. Hydrolysates of whey proteins from all animal species studied could be an interesting approach to add value to this by-product.

## 1 Introduction

Whey is currently an underutilized by-product of cheese production. Its high organic load makes it an economic and environmental issue for the dairy industry, as it cannot be returned to nature. However, its composition of proteins, lactose, vitamins, and minerals, which can be extracted using various separation methods, particularly emphasized its potential. After extraction, the properties of these molecules, especially the proteins, are utilized in various areas such as food, pharmaceuticals, cosmetics, or energy production (1). The qualitative and quantitative distribution of soluble proteins depends on the animal species. Cow’s milk is the most studied milk in the world, as it dominates global milk production. Various proteomic approaches have been used to characterize the whey proteins of cow’s milk in order to learn more about these proteins, particularly in view of the hypersensitivity reactions to β-Lg, which mainly affect infants and can persist into adolescence (2). β-Lg and α-lactalbumin (α-La) are the most important protein fractions of bovine whey, but there are other whey proteins such as lactoferrin (Lf) and lactoperoxidase which play only a minor role but have been very well studied. The protein content of goat and sheep whey is quite similar to that of cow whey. The whey fraction consists of β-Lg, α-La, immunoglobulins (IgGs), bovine serum albumin (BSA) and less important proteins such as lysozyme and Lf.

Camel milk is known for its interesting medicinal and nutritional values, which are partly due to its protein composition, which differs from that of other milks. Camel milk is characterised by the presence of some specific proteins, such as whey acidic protein (WAP), Peptidoglycan recognition protein (PGRP) and whey basic protein (CWBP) and IgGs (3, 4) compared to the other milks, and the presence of these proteins gives it additional nutritional benefits. Also, camel whey is free of β-Lg, which makes it similar to human milk and can be used as a substitute for infants with allergies to this protein (3).

The proteins of mare’s whey are similar to those of cow’s whey when it comes to a qualitative assessment, with β-Lg and α-La found as dominant amounts, and IgGs, serum albumin (SA), Lf and lysozyme in low amounts (5). As with mare milk, donkey milk, its closest equid counterpart, has also drawn attention to the role of its whey proteins and bioactive peptides in human nutrition (2), though comparative data on mare whey itself remain considerably scarcer.

However, whey in its liquid form is not easy to consume, so it is important to find a method to take advantage of its protein composition, and this liquid retains several nutritional properties (6). Enzymatic hydrolysis of proteins is an affordable technology for the development of high-quality products because it has a greater application potential than chemical hydrolysis and offers several advantages, including a fast reaction rate and high specificity (6). In addition, enzymatic hydrolysis is carried out under mild conditions resulting in minimal changes to the proteins and producing food-grade hydrolysates (7). Industrial enzymes and combinations of different proteinases are used, including the gastrointestinal enzymes chymotrypsin, pepsin and trypsin or enzymes from bacterial and fungal sources (8). In addition to their high nutritional value, milk whey proteins are precursor molecules of biologically active peptides that can modulate important functions of the body (9). Indeed, the inactive peptides are encoded in the protein sequence, which become biologically active when they are released from the protein matrix and are therefore called bioactive peptides. They can be released during digestion by endogenous enzymes or even during lactic acid fermentation by microbial enzymes and also by *in vitro* proteolysis with enzymes (10). These protein fragments can indeed have a positive effect on a physiological state or function. Therefore, a health-promoting effect is achieved through this hydrolysis process with effects on the nervous, cardiovascular, immune, and digestive systems (11). Peptides derived from whey proteins play a regulatory role in oxidative metabolism, which is important for cell survival and induces oxidative changes. In addition, the enzymatic hydrolysis of whey proteins, especially from cow’s milk and to a lesser extent from ewe and goat’s milk, has been extensively researched to obtain bioactive peptides with strong antioxidant properties. Some antioxidant peptides derived from camel milk proteins like lactophorin, kaliocin, GlyCAM-1 and PGRP-1 have been identified (12). The antioxidant peptides are influenced by other factors such as the type of protease, peptide structure, degree of hydrolysis and peptide concentration (13).

Although several studies have characterised whey proteins and their hydrolysis-derived bioactivities in individual dairy species (3, 9, 10, 14), direct comparisons among species remain limited because the available data were generally generated using different experimental conditions, hydrolysis procedures, and analytical methods. Furthermore, while cow and camel whey proteins have been extensively investigated (3, 12–15), information regarding ewe and goat whey proteins (9, 16), and especially mare whey proteins (5, 17, 18), remains scarce. To our knowledge, this is the first study to simultaneously compare the physicochemical characteristics of milk and whey obtained from a cow, camel, ewe, mare, and goat under a single standardized experimental protocol and to evaluate the impact of sequential simulated gastrointestinal digestion on the antioxidant properties of their whey proteins. This approach provides a more reliable cross-species comparison and contributes new insights into the valorisation potential of whey from different dairy species.

The objectives of this study were to compare the physicochemical properties of cow, camel, ewe, mare, and goat milk, along with their respective whey. Additionally, the study examined how the enzymes pepsin and pancreatin affect the antioxidative activity of whey proteins from these five species.

## 2 Materials and methods

### 2.1 Samples preparation

Camel and goat milks were sourced from the experimental station of the livestock and wildlife laboratory of Arid Lands Institute of Medenine (Tunisia). The cow, the ewe and the mare milks were purchased from facility situated in Djerba (Tunisia).

### 2.2 Physico-chemical analysis

#### 2.2.1 pH and Dornic acidity

The pH-value was measured at 20 °C using a Jenway pH meter (Model 3,510, Staffordshire, United Kingdom) previously calibrated (19). The acidity was determined by titrating 10 mL of milk or whey with a solution of sodium hydroxide NaOH (0.1 N) in the presence of phenolphthalein (0.5%). The acidity is expressed in degree Dornic (°D) (20). Density determination was performed using lactometer, graduated at 20 °C.

#### 2.2.2 Viscosity

The viscosity was determined using a Brookfield type viscometer (Model DV-E, MA, USA) measured at 20 °C, using the rotating mobile number 3 (RV/HA/HB- 4) and a speed of 100 rpm. The viscosity is expressed in centipoise (cP).

#### 2.2.3 Color

The color parameters was determined using a colorimeter (Konica Minolta Chroma Meter CR-400/410). The CIELab color parameters (L*, a*, b*) specify different color intensities: L* value: quantification from 0 (black) to 100 (white), a* value: quantification from −100 (green) to + 100 (red) and b* value: quantification from −100 (blue) to + 100 (yellow).

#### 2.2.4 Dry matter and ash

Dry matter and ash were determined prospectively after drying of whey samples at 105 °C and incineration of the dry matter at 550 °C (21).

#### 2.2.5 Minerals

Minerals were analyzed According to ISO 8070 (2007), protocol that specifies a flame atomic absorption spectrometric method for the determination of calcium, sodium, potassium and magnesium.

#### 2.2.6 The lactose

Lactose content was determined according to the method of Dubois et al. (22) and proteins were determined by the Kjeldahl method (21).

### 2.3 Whey protein separation

Whey proteins of different milk samples were prepared as described by Lajnaf et al. (23) with some modifications. The collected samples of fresh milks were skimmed by centrifugation (1,000 × *g*, 15 min, 4 °C). Then, the samples were coagulated with animal rennet (Strength 1:16000), incubated for 1 h at 37 °C. Once separation was achieved, the whey was filtered with Whatman paper. The obtained filtrate was centrifuged again (5,000 × *g*, 20 min, 20 °C) to eliminate all the remaining para-casein, recover the whey of each species and stored at −20 °C until further analysis.

### 2.4 Enzymatic hydrolysis of proteins

Whey samples were digested, *in vitro*, according to the protocol adopted by Jrad et al. (24). In order to mimic the gastrointestinal digestion, the pH of all whey samples was gradually brought to 2.0 by the addition of 1 M HCl and pepsin (Pepsin crystalline 3,260 Units/mg Protein; Sigma Aldrich Co., St. Louis, MO, USA), previously prepared in HCL, was added at an enzyme to-substrate (E/S) mass ratio of 1/200. After homogenization, the mixture was incubated at 37 °C for 30 min with stirring. Afterwards, 30% (w/w) NaOH was added and the pH of the samples reached a value of 7.5, thus stimulating duodenal digestion. To simulate the conditions of intestinal digestion, the solutions are incubated at 37 °C for 4 h after adding pancreatin (from pancreas, activity is equivalent to 8 USP.; Sigma-Aldrich), previously prepared in deionized water, to reach an E/S ratio corresponding to 1/400. The hydrolysis reaction is stopped by incubation at 85 °C for 5 min.

### 2.5 Peptide concentration and degree of hydrolysis

Degree of hydrolysis (DH) was analyzed using the OPA method as described by Nielsen and Petersen (25) with some modifications. The OPA reagent was freshly prepared by mixing 25 mL of sodium tetraborate buffer (100 mM; pH 9.3), 2.5 mL of SDS (20%, wt/wt), 40 mg of OPA (dissolved in 1 mL of methanol), and 100 μL of β-mercaptoethanol. Final volume was raised to 50 mL with milli-Q water (Elix-10, Millipore, Molsheim, France). Small aliquots (100 μL) of the samples were added directly to the cuvette containing 1 mL of OPA reagent and mixed gently for 5 s. The absorbance was measured at 340 nm using a Spectrophotometer UV- Visible (Cecil CE 2041, Cambridge, UK) after 2 min of incubation in the dark at room temperature. Degree of hydrolysis was calculated as shown in Equation 1:


$$H\;(\%)=\frac{h}{\text{htot}}\times 100\;\;$$ (1)

Where h_tot_ is the total number of peptide bonds per protein equivalent and h is the number of hydrolyzed bonds and the number of hydrolyzed bonds was determined as follows (Equation 2):


$$h=\frac{\left(\text{Leucine}-\mathrm{NH}2-\beta \right)}{\alpha }$$ (2)

where *α*, *β*, and *h* to *t* values were 1.039, 0.383, and 8.2 mEq.g^−1^ of protein, respectively (26).

### 2.6 Electrophoresis analysis

The electrophoresis in polyacrylamide gel in the presence of sodium dodecyl sulfate (SDS-PAGE) was assessed according to Laemmli and Favre (27). Electrophoresis was performed in 5% (w/v) polyacrylamide stacking gel at pH 6.8 and in 15% w/v polyacrylamide resolving gel at pH 8.8. Samples (2 g.L^−1^) were loaded on the gel after that the protein bands were stained with Coomassie Brilliant Blue R-250. In addition, these are denatured 3 min at 100 °C in 30 μL of sample buffer. The samples are separated by polyacrylamide gel electrophoresis: with a concentration of 5% for the concentration gel, and 15% for the separation gel. A volume of 10 μL of each sample was deposited on the gel, the separation was performed at 200 V, 80 mA for 30–45 min. The migration buffer used was 50 mM Tris, 0.384 mM glycine and 0.1% (w/v). Protein revelation is done with Coomassie blue 0.1% (w/v) mixed with 2% (w/v) trichloroacetic acid and 50% (v/v) ethanol. The discoloration is done by successive washes in baths consisting of a mixture of 30% (v/v) ethanol and 10% (v/v) acetic acid in order to obtain a clear background. The gels are then dried and stored between two sheets of Cellophane.

### 2.7 Antioxidant activities

#### 2.7.1 DPPH radical-scavenging activity

The DPPH (2,2- diphenyl-1-picrylhydrazyl) radical scavenging activity was measured by the method described by Oussaief et al. (14) with some modifications. Briefly, a volume of 30 μL of each sample was added to 270 μL of ethanol solution of DPPH (125 μM) and incubated for 1 h in the dark at room temperature. The absorbance was measured at 517 nm using a microplate spectrophotometer (Thermo Scientific Multiscan Go). The control was performed in the same way, except that distilled water was used instead of the sample. Ascorbic acid was used as a positive control. DPPH radical scavenging activity was calculated as presented in Equation 3:


$$\text{DPPH radical inhibition}\;(\%)=\frac{\left[(\text{Acontrol}-\text{Asample})\right]}{\text{Acontrol}}\times 100$$ (3)

Where: *A*_control_ and *A*_sample_ are the respective absorbances of the control and sample at 517 nm.

#### 2.7.2 ABTS radical-scavenging activity

The radical scavenging activity of ABTS (2,2′-azinobis [3 ethylbenzothiazoline-6- sulfonic acid]) was determined by the method described by Jrad et al. (24) with minor modifications. ABTS·^+^ radical cation was obtained by dissolving 7 mM of the ABTS^+^ in 2.45 mM of potassium persulfate. This mixture was kept in the dark for 14 h at room temperature. The ABTS·^+^ radical cation was then diluted with sodium phosphate buffer (5 mM, pH 7.4) to obtain an absorbance of 0.70 ± 0.05 at 734 nm. Each sample (150 μL) was added to 150 μL of ABTS·^+^ radical reagent and then, incubated in the dark for 10 min at room temperature. The absorbance was measured at 734 nm using a microplate spectrophotometer (Thermo Scientific Multiscan Go). The control was performed in the same way as the sample except that the sample was replaced by distilled water. Ascorbic acid was used as a positive control. The percentage of ABTS radical scavenging activity was calculated using the following formula (Equation 4):


$$\text{ABTS radical inhibition}\;(\%)=\frac{\left[(\text{Acontrol}-\text{Asample})\right]}{\text{Acontrol}}\times 100$$ (4)

Where: *A*_control_ and *A*_sample_ are the respective absorbances of the control and sample at 734 nm.

#### 2.7.3 Ferrous ion chelating activity

The chelation of ferrous ions Fe^2+^ was determined by the protocol adopted by Oussaief et al. (14), with some modifications. A volume of 277.5 μL of each sample was added to 7.5 μL of FeCl_2_ solution (2 mM) and 15 μL of a ferrozine solution (5 mM). The absorbance was measured at 562 nm using a microplate spectrophotometer (Thermo Scientific Multiscan Go).

The control was prepared using distilled water instead of the sample. EDTA (ethylene diamine tetra acetic acid) was used as a positive control. The chelation power was calculated as follows in Equation 5:


$$\text{Chelating power}\;(\%)=\frac{\left[(\text{Acontrol}-\text{Asample})\right]}{\text{Acontrol}}\times 100$$ (5)

Where: *A*_control_ and *A*_sample_ are the respective absorbance of control and sample at 562 nm.

### 2.8 β-carotene assay

β-carotene essay was determined by the method of Koleva et al. (28). Chloroform (1 mL) was added to β-carotene (0.5 mg) and mixed with 25 μL of linoleic acid and 200 μL of Tween-80. The chloroform was completely evaporated in a rotator evaporator at 40 °C. Deionized water was then added, and the mixture was vigorously stirred. Then 150 μL of sample were added to 150 μL of the β-carotene-linoleic acid emulsion. The plate was incubated at 50 °C for 60 min. The absorbance was measured at 470 nm using a microplate spectrophotometer (Thermo Scientific Multiscan Go). The control was performed in the same way, except that distilled water was used instead of the sample. The percentage of this activity was calculated as follows (Equation 6):


$$\text{Antioxidant activity}\;(\%)=[1-\frac{\left(A0-\mathrm{At}\right)}{\left(A^{\prime }0-A^{\prime }t\right)}]\times 100$$ (6)

Where, *A*_0_ and *A*_t_ are the respective absorbances of the sample recorded before and after incubation and *A*′_0_ and *A*′_t_ are the absorbances of the control recorded before and after incubation, respectively.

All assays are performed at concentration of 1.5 mg.L^−1^ of indigested samples and 1.5 μM. Eq NH_2_ of digested samples.

### 2.9 Statistical analysis

All experiments were done in triplicate, and data were analyzed using XLSTAT (Addinsoft, 2019). A one-way ANOVA followed by Tukey’s test was used to check for significant differences among the means, at *p* < 0.05 considered statistically significant.

## 3 Results

### 3.1 Physico-chemical composition and mineral profile of the different milks and wheys

Table 1 presents the physico-chemical composition of camel, goat, cow, ewe and mare milk. The results indicate that camel milk has the most acidic (pH = 6.49 ± 0.01 and acidity = 18.45°D ± 0.51) while mare milk is the most alkaline, exhibiting the lowest acidity (pH = 7.96 ± 0.01 and acidity = 7.6°D ± 0.06). Ewe milk is the most viscous and has the highest dry matter (157.53 g.L^−1^ ± 0.52) and protein (45.85 g.L^−1^ ± 1.2) content, which contributes to its thicker texture. Mare milk, on the other hand, contains the highest concentration of lactose (59.5 g.L^−1^ ± 0.07). In terms of color, camel milk is the lightest (high L*), whereas ewe milk is the darkest among the samples.

**Table 1:** The average values of the main milk component from five animal species.

Kind of milk		Camel	Goat	Ewe	Mare	Cow
pH		6.49 ± 0.01^a^	6.53 ± 0^a^	7.29 ± 0^b^	7.96 ± 0.01^c^	6.60 ± 0^a^
Acidity (°D)		18.45 ± 0.51^a^	17.05 ± 0.6^a^	11.55 ± 0.63^b^	7.60 ± 0.06^c^	16.75 ± 0.63^a^
Density		1.028 ± 0^a^	1.029 ± 0^a^	1.03 ± 0^a^	1.025 ± 0^a^	1.029 ± 0^a^
Viscosity (cP)		3.78 ± 0.06^a^	4.15 ± 0.07^a^	7.10 ± 0.14^c^	4.45 ± 0.07^b^	3.65 ± 0.07^a^
Dry Matter (g.L^−1^)		116.85 ± 0.68^b^	134.50 ± 0.7^b^	157.53 ± 0.52^c^	107.65 ± 0.22^a^	101.88 ± 2.14^a^
Ash (g.L^−1^)		8.64 ± 0.25^b^	10.16 ± 0.36^c^	10.13 ± 0.07^c^	5.53 ± 0.05^a^	9.88 ± 0.05^b^
Protein (g.L^−1^)		27.06 ± 0.05^b^	29.92 ± 0.03^b^	45.85 ± 1.2^c^	25 ± 0.07^a^	27.44 ± 0.19^b^
Lactose (g.L^−1^)		47.10 ± 0.02^b^	46.65 ± 0.49^b^	43 ± 1.41^a^	59.5 ± 0.7^c^	45 ± 1.41^a^
Color	L*	89.10 ± 0.3^c^	79.60 ± 0.1^b^	70.42 ± 0.1^a^	83.61 ± 0.1^b^	80.01 ± 0.5^b^
	a*	−0.18 ± 0.12^b^	−1.12 ± 0.36^b^	−0.72 ± 0.5^b^	−2.53 ± 0.9^a^	−2.30 ± 0.91^a^
	b*	10.55 ± 0.15^a^	15.05 ± 0.8^c^	8.37 ± 0.4^a^	12.10 ± 0.6^b^	14.85 ± 0.2^c^

Data are expressed as means ± standard deviation. Different letters indicate significant differences (*p* < 0.05).

*^a, b, c^ Mean values with different small letters within a line are significantly different by Tukey test at (*p* < 0.05).

Whey samples from the five different skimmed milk species were obtained through rennet coagulation, and their physicochemical characteristics are presented in the Table 2. The findings show that camel whey is the most acidic (pH = 6.56 ± 0.04; acidity = 16.78°D ± 0.29), while mare whey is the most alkaline, with the lowest acidity (pH = 7.98 ± 0; acidity = 7.24°D ± 0.06). Ewe whey exhibits the highest density and protein content among the samples, as detailed in Table 2. In contrast, mare whey has the highest lactose concentration (55.45 ± 0.07 g.L^−1^). Regarding color, the b* value, which indicates yellowness, is lowest in ewe whey and highest in camel and goat whey samples. Mineral concentrations for both milk and whey samples are provided in Table 3. Goat and cow milk contain significantly higher levels of calcium (>1800 mg.L^−1^) and potassium (>2000 mg.L^−1^), which supports their suitability for coagulation and cheesemaking processes. In contrast, camel milk is notably rich in zinc (7.9 mg.L^−1^) and iron (1.17 mg.L^−1^), contributing to its therapeutic value, especially for preventing deficiencies. Among the whey samples, camel whey has the highest concentrations of sodium, potassium, zinc, and iron; goat whey contains the highest level of copper; and ewe whey is richest in calcium, as detailed in Table 3.

**Table 2:** The physico-chemical composition of whey from the five different species.

Parameters		Camel whey	Goat whey	Ewe whey	Mare whey	Cow whey
pH		6.56 ± 0.04^a^	6.57 ± 0^a^	7.56 ± 0^b^	7.98 ± 0^b^	6.67 ± 0^a^
Acidity (°D)		16.78 ± 0.29^a^	15.85 ± 0.63^b^	10.87 ± 0.31^c^	7.24 ± 0.06^d^	15.3 ± 0^b^
Density		1.025 ± 0^a^	1.026 ± 0^a^	1.027 ± 0^a^	1.023 ± 0^a^	1.026 ± 0^a^
Viscosity (cP)		2.93 ± 0.07^a^	3.9 ± 0.07^b^	3.8 ± 0^b^	4 ± 0.14^b^	3 ± 0.07^a^
Dry matter (g.L^−1^)		59.99 ± 1.12^b^	62.56 ± 0.79^b^	61.53 ± 0.15^b^	93.54 ± 0.06^a^	60.03 ± 0.31^b^
Ash (g.L^−1^)		6.39 ± 0.25^d^	7.19 ± 0.62^c^	9.49 ± 0.72^a^	3.17 ± 0.09^e^	8.53 ± 0.16^b^
Protein (g.L^−1^)		19.12 ± 0.62^d^	21.79 ± 0.6^c^	30.68 ± 0.62^a^	24.01 ± 1.25^b^	17.34 ± 0.62^e^
Lactose (g.L^−1^)		43.4428 ± 0.14^b^	41.32 ± 0.11^c^	39 ± 0.24^c^	55.45 ± 0.07^a^	40 ± 0.36^c^
Color	L*	60.92 ± 0.2^b^	53.60 ± 0.12^a^	50.24 ± 0.1^a^	60.02 ± 0.02^b^	60.27 ± 0.01^b^
	a*	−2.22 ± 0.35^a^	−1.95 ± 0.63^a^	−2.76 ± 0.07^a^	−2.29 ± 0.21^a^	−2.06 ± 0.3^a^
	b*	14.65 ± 0.5^a^	14.59 ± 0.9^a^	7.22 ± 0.03^d^	8.35 ± 0.4^c^	11.10 ± 0.1^b^

Data are expressed as means ± standard deviation. Different letters indicate significant differences (*p* < 0.05).

^a–d^ in the same line means that the mean values are significantly different.

**Table 3:** Mineral contents (mg.L^−1^) in milks and wheys of different animal species.

Mineral	Camel		Goat		Cow		Mare		Ewe	
	Milk	Whey	Milk	Whey	Milk	Whey	Milk	Whey	Milk	Whey
Na	448 ± 13.0^bA^	434 ± 12.0^bA^	634 ± 10.0^aA^	230 ± 55.0^cB^	550 ± 2.0^aA^	306 ± 12.0^aB^	632 ± 1.0^aA^	364 ± 3.3^aB^	405 ± 4.0^bA^	314 ± 16.0^bA^
K	1,660 ± 30.0^bA^	1,300 ± 7.0^aA^	2000 ± 21.0^aA^	584 ± 4.5^cB^	2006 ± 5.0^aA^	548 ± 0.1^cB^	302 ± 2.4^dA^	246 ± 11.0^dA^	1,382 ± 1.6^cA^	354 ± 5.0^bB^
Ca	638 ± 8.0^dA^	400 ± 0.1^bB^	2,100 ± 3.0^aA^	362 ± 2.3^cB^	1880 ± 10.0^bA^	350 ± 9.9^cB^	410 ± 7.0^eA^	242 ± 0.8.3^dB^	1,218 ± 3.4^cA^	408 ± 9.8^aB^
Mg	58 ± 0.1^dA^	34 ± 0.51^bB^	163 ± 0.11^aA^	26 ± 1.1^cB^	100 ± 0.72^cA^	24 ± 1.6^cB^	30 ± 0.58^eA^	20 ± 0.92^dB^	110 ± 0.63^bA^	64 ± 0.52^aB^
Cu	0.52 ± 0.02^dA^	0.38 ± 0.06^dA^	1.12 ± 0.17^bA^	0.94 ± 0.09^aA^	1.56 ± 0.35^aA^	0.46 ± 0.41^bA^	0.86 ± 0.2^cA^	0.44 ± 0.05^cA^	0.64 ± 0.01^dA^	0.34 ± 0.2^dA^
Zn	7.92 ± 0.5^aA^	2.62 ± 0.03^aB^	4.00 ± 0.35^cA^	0.92 ± 0.04^dB^	6.4 ± 0.5^bA^	1.82 ± 0.54^bB^	1.38 ± 0.15^eA^	1 ± 0.06^cB^	1.7 ± 0.7^dA^	1.5 ± 0.1^cA^
Fe	1.17 ± 0.01^aA^	0.87 ± 0.04^aB^	0.41 ± 0.09^cA^	0.27 ± 0.001^cB^	0.53 ± 0.02^bA^	0.39 ± 0.04^bB^	0.32 ± 0.08^dA^	0.25 ± 0.08^dA^	0.5 ± 0.01^bA^	0.42 ± 0.03^bA^

Data are presented as means ± standard deviation. ^bA, aA, cB, aB, dA, bB, eA, cA^Mean values with different lowercase letters within the same row are significantly different according to the Tukey test (*p* < 0.05) for milks and wheys between species. Uppercase letters in the same row indicate significant differences (*p* < 0.05) for milks versus wheys within the same species.

### 3.2 Hydrolysis of whey proteins

Peptides concentration and degree of hydrolysis of ewe, goat, camel, cow and mare whey hydrolysates are presented in Table 4. The highest peptide concentration was found in the digested ewe whey sample (75.29 mM), while the lowest was observed in the digested mare whey sample (19.02 mM). Furthermore, during the simulated gastrointestinal digestion, the degree of hydrolysis (DH), which correlated with peptide concentration, ranged from 4.9 to 97.03% across all whey samples (Table 4). Specifically, the DH for goat whey proteins was 40.52%, while camel whey proteins had a DH of 23.45%.

**Table 4:** Peptide concentration and degree of hydrolysis of whey of the five species.

Samples	Peptide concentration (mM)	Degree of hydrolysis (%)
Digested ewe whey	75.29 ± 1.19^a^	97.03 ± 1.89^a^
Digested goat whey	37.66 ± 1.37^b^	40.52 ± 1.17^b^
Digested camel whey	29.69 ± 0.78^c^	23.45 ± 1.24^c^
Digested cow whey	23.61 ± 0.03^d^	11.59 ± 0.05^d^
Digested mare whey	19.02 ± 0.43^e^	4.90 ± 0.05^e^

Data are expressed as means ± standard deviation. ^a–e^Different letters indicate significant differences (*p* < 0.05).

### 3.3 Electrophoretic profile of total whey proteins from the five animal species and their corresponding hydrolysates

Figure 1 shows the protein profiles of camel, ewe, cow, mare and goat whey, along with their corresponding hydrolysates obtained by successive pepsin and pancreatin digestion.

**Figure 1:**
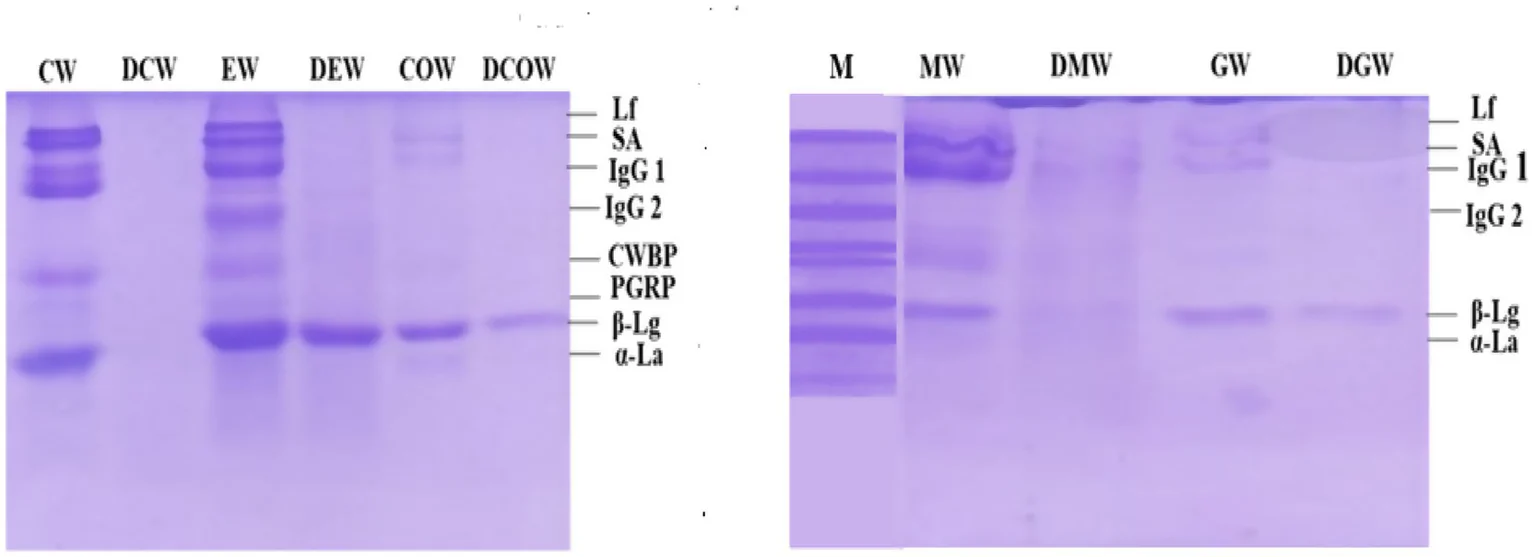
Sodium dodecyl sulphate–polyacrylamide gel electrophoresis (SDS-PAGE) of Camel whey (CW); Digested camel whey (DCW); Ewe whey (EW); Digested ewe whey (DEW); Cow whey (COW) and Digested cow whey (DCOW); Mare whey (MW); Digested mare whey (DMW); Goat whey (GW) and Digested goat whey (DGW). Lf, lactoferrin; SA, serumalbumin; IgGs, immunoglobulins; CWBP, Camel whey basic protein; PGRP, Peptidoglycan-recognition-protein; β-Lg, β-lactoglobulin; α-La, α-Lactalbumin; M, (molecular weight): Aprotinin, bovine lung (6,500); α-Lactalbumin, bovine milk(14,200); Trypsin inhibitor, soybean (20,000); Trypsinogen, bovine pancreas (24,000); Carbonic anhydrase, bovine erythrocytes (29,000); Glyceraldehyde-3-phosphate dehydrogenase, rabbit muscle (36,000); Ovalbumin, chicken egg (45,000); Albumin, bovine serum (66,000).

Five common bands, representing major whey proteins-lactoferrin (Lf), serum albumin (SA), immunoglobulin G1 (Ig-G1), immunoglobulin G2 (Ig-G2) and alpha-lactalbumin (α-La)- are detected in all whey samples. The bands corresponding to Lf and SA did not migrate further due to their high molecular weight. The band corresponding to Lf is very intense for CW and EW, moderately intense in MW, and presents a very low intensity for other whey samples. This could be explained by the different concentrations of Lf and SA in the various whey samples. Additionally, the electrophoretic profiles show that Lf is sensitive to the action of enzymatic hydrolysis because the band characterizing cow, goat, ewe, and camel Lf clearly disappears; however, this band persists with a slight intensity in digested mare whey (DMW), which is expected given the lowest degree of hydrolysis (Table 4).

### 3.4 Antioxidants activities of native and hydrolyzed whey proteins from the five animal species

This study aims to investigate the antioxidant activities. DPPH, ABTS, Fe^2+^ chelating ability, and β-caroten assays were used to determine the antioxidant activities of the various samples. The results of the DPPH and ABTS radical-scavenging activity of camel, goat, cow, mare, and ewe whey samples and their corresponding hydrolysates are presented in Figure 2. With respect to the native whey samples at the concentration of 1.5 mg.ml^−1^ of proteins, the ewe whey presents the highest DPPH radical scavenging activity (83.86%) versus 79.23, 71.83, 59.63 and 44.50% for cow, camel, goat and mare whey samples, respectively. After digestion, camel whey proteins exhibited the strongest DPPH radical scavenging activity. The native whey protein samples from cow and camel had the highest ABTS radical scavenging activity (54.16 and 48.97%; respectively) at the same protein concentration (1.5 mg.ml^−1^), but the whey proteins from ewe and mare displayed the lowest ones. For goat and mare whey, this activity is enhanced after gastro-intestinal digestion.

**Figure 2:**
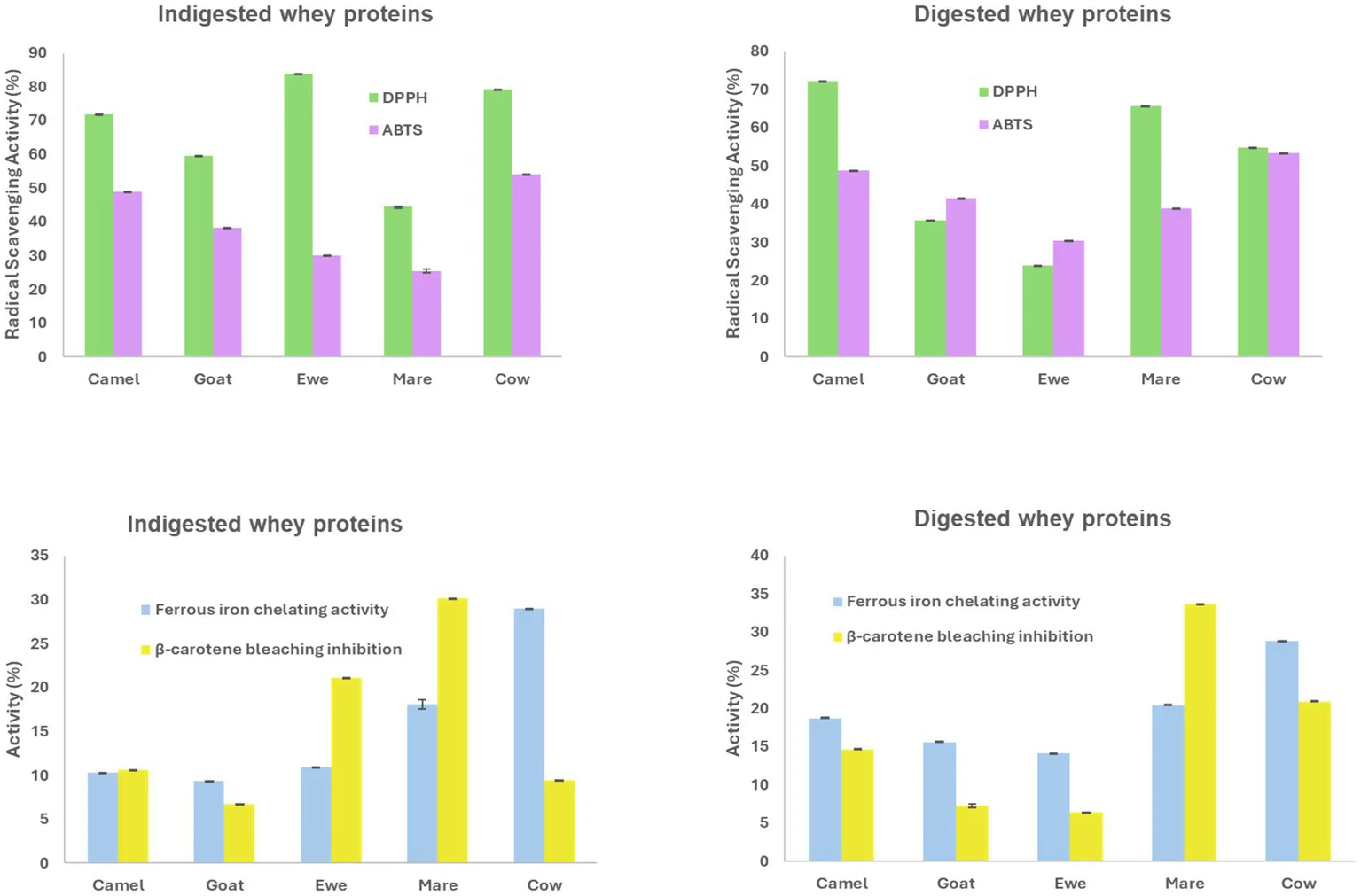
Antioxidant activity *in vitro* of the undigested and digested whey proteins from milks of five different species with four complementary tests: DPPH and ABTS radical scavenging activities, ferrous ion chelating ability and β-carotene bleaching inhibition capacity.

The antioxidant activity of both indigested and digested whey samples was evaluated using the ferrous ion chelating assay (Figure 2). The antioxidant activity of both indigested and digested whey samples was measured using the ferrous ion chelating assay (Figure 2). Before digestion, the cow whey sample exhibited the highest ferrous ion chelation activity, while goat, camel, and ewe whey proteins showed comparatively weaker metal-chelating abilities. After simulated digestion, the chelating activity increased in all whey samples, especially in camel and goat whey proteins. Cow whey proteins, however, maintained their high activity, demonstrating strong potential as effective iron chelators.

Figure 2 shows the ability of undigested and digested whey proteins to inhibit β-carotene bleaching. It reveals that mare whey proteins had the highest inhibitory activity both before and after simulated gastrointestinal digestion. Additionally, the lipid-protective capacity of cow and camel whey proteins improved after digestion.

### 3.5 Structure–activity relationship

The correlation between degree of hydrolysis (DH), peptides concentrations and antioxidant activities of different whey hydrolysates was presented in Figure 3. A high positive correlation was found between DH and peptides concentrations (*r* = 0.998, *p* < 0.0001), indicating that increased proteolysis promotes peptide release. However, DH showed a tendency toward a negative correlation with antioxidant assays (*r* = −0.69 to −0.84), though these correlations were not statistically significant (*p* > 0.05). This suggests that antioxidant activity is not directly dependent on the degree of hydrolysis or peptide concentration. Instead, antioxidant properties are more influenced by peptide sequence, molecular weight, and amino acid composition. Positive correlations were observed among different antioxidant assays, particularly between ABTS and ferrous ion chelating activity (*r* = 0.791), and between DPPH and β-carotene bleaching inhibition (*r* = 0.708), suggesting that some peptides may contribute to multiple antioxidant mechanisms simultaneously.

**Figure 3:**
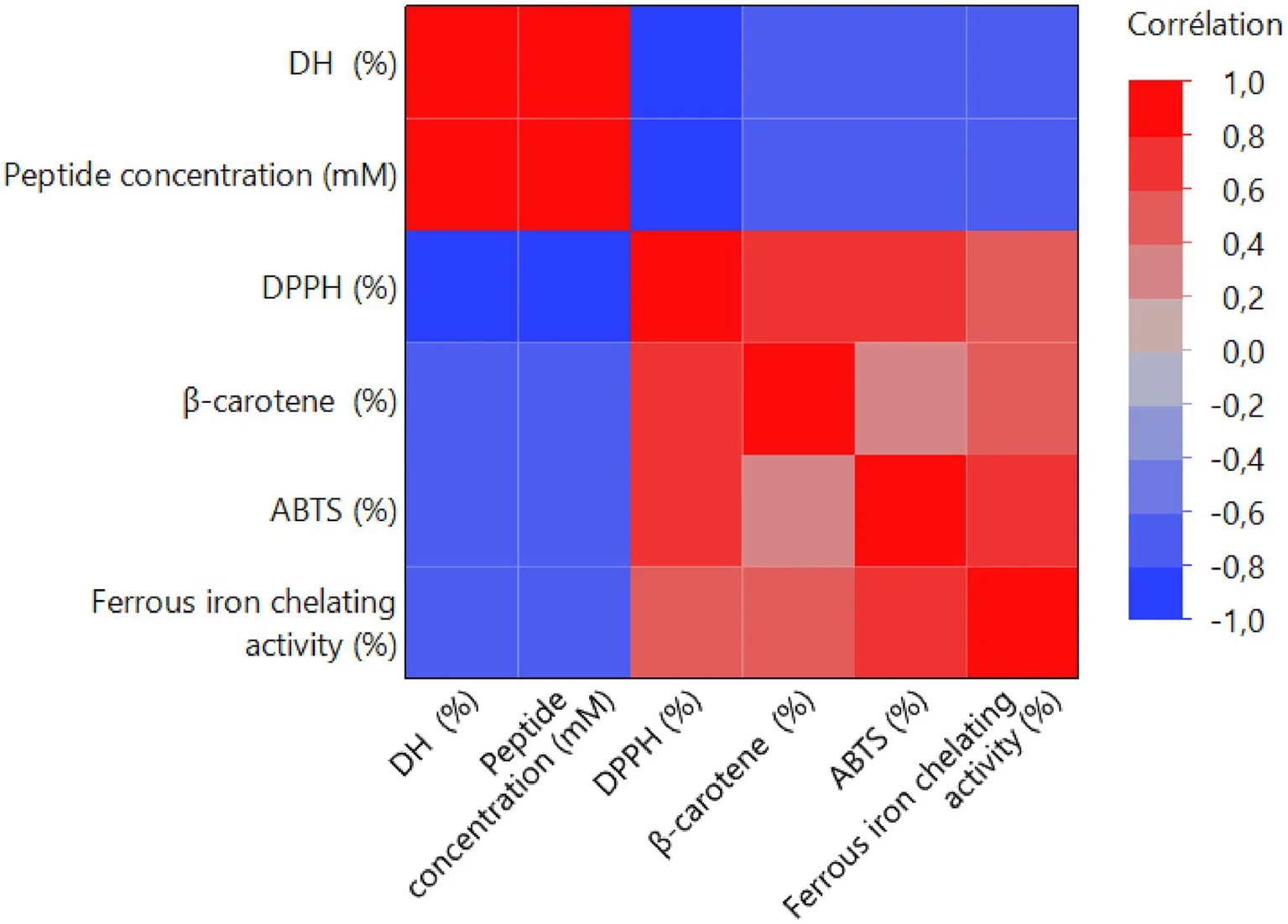
Correlation between degree of hydrolysis (DH), peptide concentration and antioxidant activities.

## 4 Discussion

The comparative analysis of milk components from different dairy species revealed that camel milk is the most acidic, confirming findings by El-Hatmi et al. (29). Ewe milk presents the highest value of density 1.03 ± 0 which is lower than the value of 1.034 reported by Park et al. (30), as well as the highest protein content and dry matter as shown in Table 1. Mare milk had a significantly higher lactose concentration, which is in accordance with the results reported by Bornaz et al. (16). Color coordinates a* and b* indicate that mare milk tend toward green, while cow milk leans toward yellow. Furthermore, camel milk was significantly brighter (highest L* value) than the other milks studied, likely due to the unique structure and content of its fat globules and casein colloidal particles. Moreover, b* values, which correspond to the yellowness, were lowest in ewe milk and highest in goat milk. This difference could be because β-carotene is converted to retinol in ewe milk, resulting in an uniform white milk, whereas cow, goat and mare milk have a yellowish color caused by the presence of carotenoids (31).

After milk coagulation, a slight increase in pH-values was observed in all whey samples, caused by the release of the high negatively charged caseinomacropeptide (CMP), which neutralizes protons. On the other hand, density, viscosity, dry matter and ash content all showed a slight decrease in whey samples compared to the original milks. Protein concentrations aligned with those observed by studies of Al-Shamsi et al. (15) and Markiewicz-Keszycka et al. (17). Furthermore, lactose content was lower in whey than in milk across all five species, likely due to some lactose being retained in the coagulum. Finally, the brightness of whey samples decreased compared to milk, a change attributed to the removal of para-casein and the presence of vitamins in the soluble fraction (32).

Major and trace minerals play essential roles in the physiology and metabolism of organisms. Table 3 shows the levels of Na, K, Ca, Mg, Cu, Zn and Fe in camel, goat, ewe, mare and cow milk and whey. These results align with previous studies (2, 9, 18). Goat’s milk is the richest in macro-elements (Ca, Na, K, Mg), highlighting its high nutritional value and digestibility. Camel’s and mare’s milk have lower calcium levels (~400–600 mg.L^−1^), which contributes to their more fluid texture and makes their coagulation slower and more difficult. Additionally, whey contains fewer minerals than the original milk. This is because Na and K are almost entirely soluble in whey, while Ca and Mg remain partially bound in the colloidal suspension of casein micelles (8).

During enzymatic hydrolysis of proteins, both peptides and free amino acids were released, which is of a great interest in the human nutrition due to their potential nutritional and biological benefits (33). Camel milk whey proteins achieved a significant DH-value, consistent with previous findings on the hydrolysis of camel whey proteins using chymotrypsin (33). In this study, the DH of cow whey proteins was about 11.59%, which is lower than the results (DH = 26.6%) obtained by Silva (34).

The sensitivity of whey proteins to hydrolysis varies between milk types. For examples, camel lactoferrin (Lf) is particularly sensitive to enzymatic hydrolysis due to multiple cleavage sites for the enzymes studied, as reported by Jrad et al. (35). SDS-PAGE profiles show that serum albumin (SA) proteins of cow, ewe, goat and camel milk aren’t resistant to hydrolysis, whereas mare SA shows a band with low intensity, suggesting partial digestion. In addition, the IgGs bands from all whey samples have the same profile. Two specific serum proteins unique to camel milk - CWBP and PGRP-were detected only in camel whey but disappeared after enzymatic hydrolysis.

The electrophoretic profile of camel whey proteins shows no bands homologous to β-Lg. In contrast, β-Lg bands from cow, ewe, goat and mare decreased in intensity during hydrolysis, which is in accordance with Salami et al. (36). Eventually, the α-La band was more intense in camel whey compared to other species, where it was nearly invisible. This study reveals that α-La disappeared after digestion in all species, confirming its sensitivity to enzymatic hydrolysis. Salami et al. (36) reported that bovine α-La is completely hydrolyzed after 5 h of hydrolysis, whereas camel α-La is fully hydrolyzed within 30 min. Furthermore, α-La has shown attractive nutritional and biological properties due to their richness in essential amino acids and their antioxidant and anti-tumor activities (2). In conclusion, this study demonstrates that Lf is the most sensitive serum protein to the simulated gastrointestinal digestion, followed by α-La while β-Lg was the most resistant.

Alternatively, producing hydrolysates is a valuable way to add value to whey, as both whey and its enzymatic hydrolysates display biological activities often linked to bioactive peptides. Radicals such as DPPH and ABTS are unstable compounds that stabilize by accepting electrons or hydrogen, making them common substrates for measuring the antioxidant activities (14). Among the digested whey proteins, camel whey hydrolysates presented the highest DPPH radical scavenging activity (72.23%) compared to the other samples, indicating the release of radical scavenging peptides. This result is close to that reported by Oussaief et al. (14) for whole camel milk protein hydrolysates (68.33%). However, Al-Shamsi et al. (17) found higher radical scavenging activity in pepsin and other proteolytic enzymes-hydrolyzed whole goat milk compared to camel milk. These findings suggest that gastro-intestinal digestion affect antioxidant properties differently based on the protein composition of different whey types. The enhanced activity seen in camel and mare whey proteins may be due to the release of low-molecular weight peptides containing antioxidant amino acid residues such as tyrosine, tryptophan, histidine, methionine, cysteine and proline, known for their hydrogen or electron donating abilities (36).

The ABTS radical scavenging activity assay revealed a distinct pattern of response. Despite lower ABTS scavenging activity in undigested samples compared to DPPH activity, digestion increased the radical activity of goat and mare whey proteins. Differences obtained between ABTS and DPPH assays could be due to the fact that DPPH evaluates mainly hydrogen atom transfer in organic solvents like ethanol which makes the assessment of hydrophilic antioxidants difficult, whereas ABTS can react with both hydrophilic and lipophilic antioxidants through transfer electron mechanisms (36). Therefore, peptides generated during gastrointestinal digestion may exhibit different affinities depending on the radical system used.

Transition metal ions like Fe^2+^ accelerate the generation of reactive oxygen species through the Fenton reaction which speeds up the oxidation process. Therefore, the assessment of ferrous chelating activity is an important measure of antioxidant properties (14). Generally, results from this experiment show that the *in vitro* hydrolysis by digestive proteolytic enzymes of whey proteins increased their ferrous ion chelation ability for all the species except cow whey, which was not affected. This finding could highlight the generation of bioactive peptides rich in acidic amino acids (e.g., Glu and Asp), which are capable of chelating this metal ion (37). It was reported that the improvement of metal-chelating activity of milk proteins after hydrolysis can be explained by a better accessibility of the antioxidant residues to the metal ion and a better contribution of lower-molecular-weight peptides in chelating metal ions (38). The evaluation of antioxidant activity using the β-carotene bleaching test is based on the measurement of conjugated diene hydroperoxides resulted from the oxidation of linoleic acid. It is known for many years that carotenoids undergo “bleaching,” for example their color disappears when exposed to radicals or oxidizing species (24). Clearly, camel, goat, cow, and mare hydrolysates exhibited higher β-carotene bleaching inhibition than their undigested whey, which confirms the liberation of bioactive peptides with increased amphiphilic properties, allowing better interaction with lipid substrates and more efficient inhibition of oxidative chain reactions. In general, the results indicate that gastrointestinal digestion affects the antioxidant activities of whey proteins in a species-dependent manner. While digestion enhanced certain antioxidant mechanisms, it decreased others, reflecting differences in peptide sequences released from each whey protein source. Such variability is very related to the intrinsic composition of whey proteins, like β-Lg, α-La, SA and Lf, whose relative abundance differs among animal species. Indeed, the work of Salami et al. (39) reported a positive correlation between antioxidant activity of whey and its content of α-La and this activity is not only attributed to the quantitative factor where the quality of content plays a determining role in this biological activity. In fact, bioactivities are largely dependent on the protein to be hydrolyzed, the various cleavage sites, the diverse types of peptides produced with distinct amino acid sequences, molecular weights and structural properties (14).

Despite having the highest DH, digested ewe whey did not show the highest antioxidant activity in every assay. In the DPPH assay, camel whey hydrolysates displayed the greatest scavenging activity (38.56%) after digestion, even though camel DH (23.45%) was less than ewe whey, suggesting that the specific peptide sequences released from camel whey were primarily responsible for this activity. This is consistent with the compositional particularities of camel whey, which lacks β-Lg and instead contains CWBP and PGRP, both of which disappeared after hydrolysis (Figure 1) and whose released fragments may be intrinsically more effective radical scavengers. Similarly, camel α-La has been reported to be fully hydrolyzed within 30 min, compared with up to 5 h for bovine α-lactalbumin (36), indicating faster release of potentially bioactive fragments from the camel protein matrix even at a comparatively moderate overall DH.

The ferrous-ion chelating assay showed a different pattern again: cow whey, which had a low DH (11.59%), displayed the strongest chelating activity both before and after digestion. This suggests that metal-chelating capacity in cow whey depends more on the intrinsic amino acid composition and specific binding residues of the native and lightly hydrolyzed proteins than on extensive peptide release, consistent with reports that chelation is largely governed by residues (e.g., histidine, aspartate, glutamate) rather than peptide abundance, though further analysis, such as amino acid sequencing, is needed to confirm this.

The most striking dissociation between DH and activity was observed in the β-carotene bleaching assay, where mare whey, despite showing the lowest DH (4.90%) and the lowest peptide concentration of all species, exhibited the highest inhibition of β-carotene bleaching (72.28%). This indicates that the small number of peptides released from mare whey under the conditions tested was nonetheless highly effective in this lipid-based assay, possibly reflecting a favorable hydrophobicity profile suited to protecting the lipid-emulsion system, even in the absence of extensive proteolysis. By contrast, both ewe and cow whey showed a decrease in β-carotene bleaching inhibition after digestion relative to their native form, suggesting that, for this specific assay, the peptides generated in these two species lacked meaningful lipid-protective activity, or that extensive hydrolysis (as in ewe whey) may have degraded pre-existing antioxidant epitopes present in the native protein.

Overall, these assay-specific and species-specific patterns indicate that antioxidant activity in whey hydrolysates is governed by a combination of factors: degree of hydrolysis, the specific proteins present in each species whey, their differential susceptibility to gastrointestinal enzymes and the physicochemical properties (e.g., hydrophobicity, charge) of the resulting peptides rather than by DH alone. Peptide sequencing and molecular weight fractionation of the hydrolysates would be needed to identify the specific structural features driving these differences and were beyond the scope of the present study but represent a valuable direction for future work.

## 5 Conclusion

This study is a comparative evaluation of whey proteins from five mammalian species before and after simulated gastrointestinal digestion, providing a comprehensive overview of their antioxidant properties. Among the physicochemical findings, camel milk and whey were the most acidic of the five species (pH 6.49 and 6.56, respectively) and were comparatively rich in zinc and iron. Ewe’s milk had the highest protein content (45.85 g.L^−1^), viscosity and dry matter among the species studied, consistent with its established suitability for cheesemaking, while mare’s milk was more alkaline, lower in protein and minerals, but richer in lactose. Minerals were consistently lower in whey than in the corresponding milk across all species, likely reflecting the differential partitioning of minerals such as Ca and Mg toward the casein micelle fraction. SDS-PAGE analysis confirmed a broadly conserved whey protein profile across species, with camel whey the clear exception: it lacked β-lactoglobulin and carried two additional species-specific proteins (CWBP and PGRP), a compositional feature consistent with previous reports. Simulated gastrointestinal digestion generated peptides with antioxidant activity in all five species, supporting the premise that whey hydrolysates represent a promising route for valorizing this by-product. However, this activity was clearly assay- and species-dependent: ewe whey showed the highest native DPPH-scavenging activity, camel whey hydrolysates performed best after digestion in the same assay, cow whey was the strongest ferrous-ion chelator, and mare whey hydrolysates were most effective at inhibiting β-carotene bleaching. No single species outperformed the others across all four assays, underlining that antioxidant potential in whey is multidimensional and cannot be reduced to a single ranking. Taken together, these findings, obtained through a combination of physicochemical, mineral, electrophoretic, digestibility and antioxidant analyses, point to potential relevance for functional food development and for the utilization of dairy by-products, particularly through the generation of species-specific antioxidant hydrolysates. At the same time, some limitations should be kept in mind when interpreting the results, the *in vitro* digestion protocol, while standardized and informative for cross-species comparison, cannot fully reproduce the variability of human gastrointestinal digestion *in vivo*- particularly the influence of gut microbiota composition and individual physiological differences that may affect the actual bioavailability of the minerals and peptides observed in this study. Additionally, the four antioxidant assays used are chemical, not physiological, readouts of activity. Therefore, our results should be interpreted as a comparative basis, but further work, including peptide identification and characterization, larger-scale sampling, and validation in cellular or *in vivo* models, would be needed before the functional potential highlighted here can be translated into concrete food or nutraceutical applications.

## Data Availability

The original contributions presented in the study are included in the article/supplementary material, further inquiries can be directed to the corresponding author.
